# Immunoelectrochemical assessment of human IgE in non-invasive samples of allergic individuals using PdNCs-labelled antibodies

**DOI:** 10.1007/s00604-025-07083-3

**Published:** 2025-03-18

**Authors:** Alejandro Rodríguez-Penedo, Estefanía Costa-Rama, Rosario Pereiro, Beatriz Fernández, M. Teresa Fernández-Abedul

**Affiliations:** Department of Physical and Analytical Chemistry, University of Oviedo, Julian Clavería 8, 33006 Oviedo, Spain

**Keywords:** Immunoelectroanalysis, Linear sweep voltammetry, Elemental mass spectrometry, Palladium nanoclusters, Non-invasive samples, Allergy, IgE quantification

## Abstract

The escalating global prevalence of allergies presents a substantial public health challenge. Immunoglobulin E (IgE) serves as a key biomarker for allergic diseases, often measured in blood serum by ELISA immunoassays. Despite recent interest in minimally invasive sampling of biological fluids, the low sample volumes and IgE concentrations demand highly sensitive methodologies, typically confined to centralized laboratories. In this article, a decentralizable approach based on competitive immunoassays using Pd nanocluster (PdNCs)-labelled antibodies for electrochemical detection is proposed. With this aim, PdNCs were successfully bioconjugated with an anti-hIgE antibody to perform competitive immunoassays. To improve the analytical capabilities of the methodology, disposable screen-printed carbon electrodes with dual working electrodes were used for enhancing precision. Prior electrodeposition of PdNCs at − 0.6 V for 90 s significantly improved sensitivity (7.1 µA g ng⁻^1^) and lowered the limit of detection (LoD) to 0.3 ng g⁻^1^ for PdNCs determination. The use of PdNCs as labels resulted in an improvement in the LoD for IgE determination. Calibration curves performed using competitive immunoassays for IgE determination, ranging from 10^−5^ to 10^2^ ng g^−1^, demonstrated sensitivity comparable to high-tech methods, with a LoD of 0.008 ng g^−1^ for electrochemical measurements. Bimodal detection of Pd (linear sweep voltammetry and inductively coupled plasma–mass spectrometry) in various biological fluids (saliva, tears, nasal exudate, capillary blood, and blood serum) revealed significant differences in IgE levels between allergic and non-allergic individuals. Notably, capillary blood correlated strongly with serum blood, and a certain correlation has also been found with nasal exudate. The electrochemical approach, combining sensitivity and precision with non-invasive sampling, offers a simplified alternative for IgE determination in allergic disease.

**Graphical Abstract:**

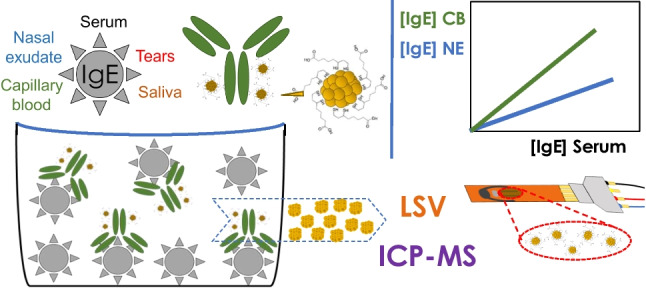

**Supplementary Information:**

The online version contains supplementary material available at 10.1007/s00604-025-07083-3.

## Introduction

Allergies have become more noticeable on a global scale, presenting a significant challenge to public health. The complex interplay between genetic and environmental factors has contributed to this increase, particularly allergic rhinitis [1]. Apart from its prevalence, better diagnostics have probably also aided to this increase. Within this context, it is known that immunoglobulin E (IgE), a class of human antibodies involved in allergic responses, plays a crucial role in the pathogenesis of e.g., rhinitis [2], asthma [3], or atopic dermatitis [4]. Thus, it can be used as biomarker for diagnosis and prediction of therapy responses in allergic diseases [5]. On the other hand, IgE has also been found to play a very important role in non-allergic airway inflammation [6]. Likewise, it appears that it could be a potential biomarker for cancer [7, 8]. Typically, IgE determination is carried out in blood serum using ELISA immunoassays, with allergy sufferers usually having values above 100 IU mL^−1^ (*ca.* 242 ng mL^−1^) [9]. Although IgE concentration is commonly measured in blood serum, in recent years there has been a growing interest in other biological fluids, as they could provide additional information about the localized allergic response, or their collection can be less invasive [10]. Thus, concentration of IgE in saliva [11], nasal exudate [12], tears [13], or capillary blood [14] has been reported, although there is no knowledge that all these samples have been studied simultaneously for the same patients previously. The use of minimally invasive samples as source of biomarkers opens the door to the decentralization of clinical analysis. However, since both the amount of sample extracted and the concentration level of IgE found in these fluids are particularly low [10–15], highly sensitive methodologies are required.

In this context, competitive immunoassays based on the use of antibodies labelled with metal nanoclusters (MNCs, abbreviation widely employed [16], as is also NCs [17]) have proven to be very sensitive [18], significantly improving the limits of detection (LoD) of conventional ELISA immunoassays [19, 20]. MNCs are small spherical groupings of metal atoms (e.g., Au, Ag, Pt, etc.) typically smaller than 3 nm in size. Their small size means that they have discrete energy levels [21], giving them physicochemical properties different to traditional nanoparticles (NPs). It has also been shown that MNCs are often superior to conventional metal nanoparticles in terms of their catalytic properties, mainly because they have a larger number of active sites together with a higher specific surface area [22]. Particularly, in the case of electrogenerated PdNPs, it has been demonstrated that the use of lower scan rate or higher cycle number increased the size and amount of PdNPs but producing a lower catalytic activity over formic acid oxidation [23]. It is difficult to prepare small-size PdNPs using electrodeposition, which would be beneficial towards achieving higher catalytic mass activity [24]. Synthetic MNCs can be coated with a layer of small thiolated organic ligands to enhance their stability and facilitate their bioconjugation with target biomolecules.

Other authors have successfully synthesized MNCs of different metals (mainly Au and Pt) that exhibit catalytic activities on various chemical reactions [25, 26]. Particularly, PdNCs have shown catalytic activity in multiple electrochemical reactions, such as oxygen reduction reaction (ORR) [27] or hydrogen evolution reaction (HER) [28]. The Volcano plot for HER [29] is used to determine the efficiency of a metal, with Pt being the most efficient, closely followed by Pd. Some authors consider Pd to be the best alternative to Pt [30], while also mentioning the usefulness of Pt with examples such as PdNPs having higher catalytic activity than Pt/C. It should be added that Pd is almost as effective as Pt at a much lower cost. The mechanism of this reaction strongly depends on the catalytic pathway [30]. It is known to work in an acidic environment, with the process occurring in three stages. The first step (also known as the Volmer or discharge step) involves the combination of a proton with an electron at the surface of the catalyst, resulting in the formation of an adsorbed hydrogen atom. The next steps depend on the pathway and can be either the Heyrovsky step (discharge reaction) or the Tafel step (recombination reaction).

PdNCs could be determined using field-deployable low-cost electrochemical readers and, also, through elemental mass spectrometry (MS), due to the presence of several hundred metal atoms per NC [31–33]. In this context, MNCs immunoprobes enable high signal amplification, allowing the detection of target biomolecules even at the low-picograms per gram levels [34]. This is not only a significant improvement in the detection limits with respect to conventional ELISA immunoassays, where enzymes are employed as labels, but also the use of electrocatalytic MNCs makes it possible to perform detection by multiple techniques in a complementary and simultaneous way.

The use of disposable, miniaturized, and low-cost screen-printed electrodes has changed the conception of electroanalytical methods, improving their analytical and productive features [35]. The versatility of the screen-printed electrochemical cells allows the easy production of cells containing more than one working electrode, which are truly useful for simultaneous determination of two or more analytes [36, 37]. A potential interesting application of these dual screen-printed cells could be the capability to record two measurements for the same assay format and analyte simultaneously, under identical ambient conditions, which could enhance the precision of the results.

In the field of electrochemical metal quantification, it is a common practice to use, when possible, a cathodic electrodeposition step [38]. This step concentrates metal atoms on the electrode surface before voltammograms are recorded, resulting in a very low LoD. Furthermore, metal nanoparticles (MNPs) that are directly electrogenerated on the working electrode have demonstrated an increase in catalytic activity, leading to higher analytical signals [39]. In this work, we have shown that the electrodeposition of PdNCs, which are used as tags of antibodies, increases the analytical signal. This enhancement is observed when the PdNCs are solvated and electrodeposited just prior to the measurement, resulting in the generation of fresh and active PdNCs on the working electrode surface, thereby increasing the catalytical effect [40]. Moreover, the proven stability and robust catalytic activity of PdNCs on HER make them a superior choice compared to traditional enzymes when tailored for decentralized analysis [41].

The combination of immunoelectrochemical approaches with miniaturized electrodes and potentiostats holds the potential for decentralized analysis (e.g., in outpatient clinics or at home), resulting in substantial improvements in patient accessibility and diagnostic speed [42, 43]. The application of these methodologies to minimally invasive samples offers an interesting alternative to conventional centralized ELISA immunoassays for IgE determination [11–14, 44]. This allows for quicker, yet precise and sensitive quantification where it is needed. In this study, we employed the developed immunoelectrochemical method along with inductively coupled plasma–mass spectrometry (ICP-MS) for analysing various samples, such as tears, saliva, nasal exudate, capillary blood, and blood serum. The goal is to expand our current understanding by exploring differences in IgE concentration while using a novel methodology focused on the use of PdNCs as bioconjugable bimodal tags that allow the determination of IgE at extremely low concentration in small volumes of biological samples.

## Experimental

### Materials and reagents

High-purity potassium tetrachloropalladate (Sigma-Aldrich) was used as the precursor salt (the salt that is used as a starting point in the synthesis of MNCs) for the synthesis of PdNCs. DL-α-lipoic acid (ACROS organic) was chosen as the ligand agent, and sodium borohydride (Sigma-Aldrich) served as the reducing agent. The basic solution was prepared using high-purity sodium hydroxide (Sigma-Aldrich). For purification, Amicon^®^ Ultra-0.5 filters with membranes of 3 kDa and 100 kDa (Millipore Amicon®) were employed. The labelling of the selected antibody with PdNCs was achieved using 1-ethyl-3-(3-dimethylaminopropyl) carbodiimide (EDC) (Across Organics) and N-hydroxysuccinimide (NHS) (Sigma-Aldrich).

For the immunoassays, the ε-chain specific anti-human-IgE antibody (anti-hIgE Ab) produced in goat (Sigma Aldrich–I2684) and a native human IgE protein (abcam–ab65866) were employed both for assessing the recognition capabilities of the Ab labelled with PdNCs and for performing competitive immunoassays to determine IgE levels in human serum, capillary blood, saliva, nasal exudate, and tears. Microtiter plates (96 well; Thermo Fisher Scientific) were used as substrate for the immunoassays and bovine serum albumin (BSA) (Merck) as blocking agent. The immunoassay procedure employed a 10-mM phosphate buffer saline (PBS) solution (pH 7.4), prepared using high-purity anhydrous disodium hydrogen phosphate and sodium dihydrogen phosphate dihydrate (both from VWR), in addition to 0.05% Tween-20 (Sigma-Aldrich). To extract PdNCs from the wells, sodium dodecyl sulphate (SDS) (Sigma-Aldrich) was used. Validation of the methodologies using PdNCs labels was performed using an IgE ELISA Kit for Human Serum (Abnova—KA0216) with spectrophotometric detection.

For ICP-MS measurements, Pd and Rh standards (analyte and internal standard, respectively) were prepared from 1000 mg L^−1^ Pd and Rh standard solutions (Sigma-Aldrich) in 2% ultrapure HNO_3_ (Merck). In electrochemical measurements, optimization studies involved the use of HNO_3_ solutions (0.1–1 M). Deionized ultrapure water with a resistivity of 18.2 MΩ cm (Purelab Flex 3&4; ELGA-Veolia) was consistently used throughout the experiments.

### Instrumentation

Electrochemical measurements were performed using a µ-Autolab Type II instrument by EcoChemie BV, Utrecht, Netherlands, controlled by the Autolab GPES software. Flexible screen-printed electrodes (SPEs, MicruX Technologies, Spain) with a counter electrode (CE) of C ink, a pseudoreference electrode (RE) of Ag ink, and one circle-shaped working electrode (WE) with 7.1 mm^2^ of area, or two elliptical-shaped WEs with an area of 2.3 mm^2^ each, were employed. A suitable box connector (MicruX Technologies) was used as interface between the SPEs and the potentiostat. Eppendorf Research^®^ plus micropipettes were used for the deposition of the PdNCs dispersion droplets on the electrodes, with a variable volume comprised between 0.5 and 10 µL.

Absorption measurements of the synthesis precursors and PdNCs dispersion were conducted using a spectrophotometer (Cary 60 UV–VIS, Agilent Technologies). For this purpose, a Suprasil quartz cuvette (model 114F-QS, Sigma-Aldrich) with an optical path of 10 mm and a chamber volume of 3 mL was employed. ICP-MS measurements were carried out using a 7900 series quadrupole ICP-MS instrument from Agilent Technologies. The operation parameters utilized for ICP-MS analysis are compiled in Table S1.

Other equipment used in this research includes an Elx800 absorbance microplate reader (Bio-Tek, USA), glass microcapillary tubes (Blaubrand intraMark) for collecting tear samples, nasopharyngeal swab (Nest Biotechnology) for nasal exudate samples, and a Touch Delica Plus lancet for the puncture necessary to extract capillary blood. To collect blood serum, Z Serum Sep Clot Activator tubes coated with microscopic silica particles were used (Vacuette, Madrid, Spain).

### Methods

#### Synthesis of PdNCs and labelling of anti-hIgE

PdNCs were synthesized following the procedure described elsewhere [33]. In many MNCs syntheses the ligand is usually added first, then the precursor salt and finally the reducing agent [45]. Thus, ligand and metal interact first, becoming the metal protected against further aggregation. However, the reducing agent and precursor salt can also be added first, and then the cluster is stabilized with the ligand [46] or, as in our case, first adding the ligand and the reducing agent [47]. Since lipoic acid has a disulphide bond, by first mixing lipoic acid with the reducing agent, the disulphide is reduced to the corresponding thiols, which could facilitate the further bonding with Pd. In brief, 0.0256 g of lipoic acid was dissolved in ultrapure water, and 240 µL of 2 M NaOH were also added. The vial underwent ultrasonic treatment for 5 min and was placed then in a 50°C water bath while vigorously shaken for additional 5 min. Subsequently, 2 mL of NaBH_4_ solution (0.36 M) was swiftly added to the mixture. After allowing the reaction to progress for 2 h, 1 mL of 15 mM K_2_PdCl_4_ was rapidly introduced, and stirred in a continued way for 6 h, maintaining a constant synthesis temperature.

Post-synthesis, PdNCs were purified through ultracentrifugation using 3 kDa membrane filters at 5500 rpm for 10 min. Finally, three additional washing steps with ultrapure water (5500 rpm for 10 min each) were performed. This purification step is crucial as otherwise there would still be lipoic acid, NaBH_4_, and K_2_PdCl_4_, which would interfere with subsequent measurements. Next, PdNCs were characterized by absorbance spectroscopy, HR-TEM, and ICP-MS measurements. The characterization by absorbance spectroscopy is shown in Figure S1, where a large difference between the signals for PdNCs, reaction blank, and palladium salt can be observed. HR-TEM images showed PdNCs with a spherical metal nucleus and FCC crystal structure. As described elsewhere [33] the average particle size determined was 2.49 ± 0.02 nm.

To generate the Ab:PdNCs immunoprobe by labelling anti-hIgE Ab with PdNCs, 100 µL of anti-hIgE (100 µg mL^−1^) were combined with 372 µL of the purified PdNCs dispersion in 10 mM PBS in an Eppendorf® tube and vortexed. Subsequently, 12 μL of a solution containing EDC and NHS were added, maintaining a molar ratio of Ab:EDC:NHS at 1:1500:1500. The mixture was left at room temperature with continuous stirring for 2 h. Following this, purification was carried out through ultrafiltration using a 100 kDa pore size filter, starting with an initial cycle at 4000 rpm for 10 min, followed by three washing steps with 10 mM PBS (4000 rpm for 10 min each). This purification step serves to remove all those PdNCs that have not been bound to antibodies, either through the formation of covalent bonds (carbodiimide method) or through weaker interactions. It is expected that the latter could be eliminated during the different steps of the immunoassay. Thus, the analytical signal obtained will mostly come from covalently bound PdNCs. To clarify the bioconjugation process, a schematic of how the bioconjugation of the PdNCs with antibodies takes place has been included in Figure S2.

The purified solution was then stored in the refrigerator until use. It is important to note that the preparation of the immunoprobe (anti-hIgE:PdNCs) was performed on the same day as the competitive immunoassay for IgE. Thus, a stability study of the conjugated immunoprobes should be conducted if they were to be used in clinical studies with many samples, or if commercial platforms were to be developed.

#### Sample collection protocol

Samples were collected from six healthy individuals (two males and four females) and six individuals diagnosed with allergic rhinitis (four males and two females) residing in the Principality of Asturias. It is important to highlight that none of the analysed individuals had other known pathologies and everyone was between 20 and 30 years old (26 ± 3 and 24 ± 3 years for healthy individuals and those with allergies respectively), except for one of the allergic individuals who was 52 years old. All samples were collected within a 3-day interval, with blood serum collection performed first (between 9:00 and 10:30 AM), followed by the collection of tears, saliva, nasal exudate, and capillary blood (between 11:00 AM and 12:00 PM). Full ethical approval was obtained from the Clinical Research Ethics Committee at the Principality of Asturias (Oviedo, Spain) (Ref. ES330440003591). The treatment and the dilution done for each type of sample are summarized in the ESM.

#### Direct competitive immunoassay using PdNCs as label

A competitive immunoassay was developed for the quantification of IgE in human assay, whose main experimental conditions have been established based on previous publications [20, 32, 33]. The optimization of the concentration of hIgE coating the well (from 2.5 to 10 µg mL^−1^) is shown in Figure S3. Thus, each well of a microtiter plate was coated with 100 µL of IgE protein standard at a concentration of 5 µg mL^−1^ and then incubated for 2 h at 37°C. After removing the excess of protein solution, any unbound binding sites on the well surfaces were blocked by incubating with a 1% BSA solution in PBS for 2 h at room temperature, followed by three wash cycles using 200 μL of 10 mM PBS containing 0.05% Tween 20. Meanwhile, in a separate Eppendorf® tube, 50 μL of the anti-hIgE:PdNCs immunoprobe (containing 6 μg mL^−1^ of anti-hIgE) was mixed with 50 μL of the protein standard or the evaluated biological human fluids and stirred for 15 min using a vortex. This mixture was then added to the pre-treated wells and incubated for an additional 2 h period at 37°C. Subsequently, the wells were thoroughly washed (four cycles of 200 µL of 10 mM PBS with 0.05% Tween 20), and the extraction of the PdNCs was carried out at room temperature for 30 min, adding 100 µL of 8% SDS to each well and shaking the plate at 700 rpm for 1 h at 37 °C. For all cases, the immunoassay was conducted in triplicate.

After extracting PdNCs, the dispersions were split into two aliquots for detection by linear sweep voltammetry (LSV) and ICP-MS. The IgE concentration was determined by LSV taking the intensity of the current resulting from the catalytic activity of PdNCs on the HER at a fixed potential as the analytical signal. Thus, 6 µL of the dispersion were directly taken for LSV analysis, and digestion of PdNCs was not required. This volume was deposited on the working electrode and left until dried. Subsequently, 40 μL of 0.75 M HNO_3_ were added for recording the LSV from 0 to − 1.5 V with a scan rate of 50 mV s^−1^, at room temperature. Additionally, the second sample aliquot was measured by ICP-MS to detect the Pd signal from the PdNCs. For conventional nebulization ICP-MS analysis, 50 μL of the dispersion were added to 100 μL of concentrated HNO_3_ to oxidize and dissolve Pd. The tubes were then placed in an ultrasonic bath and maintained for 15 min. Finally, a 1:75 dilution with deionized ultrapure water was performed to minimize the percentage of acid introduced into the ICP-MS. Measurements were conducted using external calibration with pure Pd standards with concentrations ranging from 0.05 to 5 ng g^−1^. The ^106^Pd isotope was also measured and compared with ^105^Pd, to confirm there was not any discrepancy related to its natural abundance. Rh was employed as the internal standard (measuring ^105^Pd and ^103^Rh isotopes, respectively) in a 1 ng g^−1^ concentration, to eliminate instrumental deviations.

#### Statistics

The data presented in the figures and tables are expressed as the mean value of the signal accompanied by the standard deviation (SD). To detect meaningful differences between data sets, we utilized a paired sample Student *t* test at a 95% confidence level. Prior to this analysis, we conducted an *F*-test to assess the equality of variances in both groups.

Data from competitive immunoassays were adjusted with MyAssays Ltd. software (https://mycurvefit.com/) using a four-parameter logistic (4-PL) model, following Eq. 1:

1 $$Y=d+ \frac{a-d}{1+{(\frac{X}{c})}^{b}}$$

where *a* denotes the signal at zero concentration,* b* is the gradient of the curve at the inflection point, *c* aligns with the inflection point itself, and *d* designates the signal at infinite concentration, i.e., the lowest signal response.

## Results and discussion

### Electrochemical detection of PdNCs

Decentralization is facilitated by low-cost and simple electrochemical techniques and immunoassays. However, to match the performance of high-tech centralized labs, sensitivity and precision need enhancement. This work implements strategies to improve PdNCs determination by LSV, including dual-electrode SPEs for simultaneous measurements and electrodeposition of PdNCs after suspension drying to boost catalytic activity. Although other nanoclusters (Pt, Au, Ag…) are also an option for catalysing HER, their ability, measured by the exchange current density, i.e., the rate of hydrogen evolution per surface area at the equilibrium potential [29], stands out for the Pt group metals. The representation of the exchange current as a function of the chemisorption energy shows a volcano with reactive elements to the left (Pd and Pt among other) and unreactive elements to the right (Au and Ag among others). Between Pt and Pd, the differences are not so pronounced, with the best efficiency for Pt. However, some authors consider Pd to be the best alternative to Pt [30], with examples such as PdNPs having higher catalytic activity than Pt/C. It should be added that Pd is almost as effective as Pt and new composites continue being studied [48].

In this context, the electrodeposition potential and time have been optimized by the method of one-factor-at-a-time, as illustrated in Fig. S4A and S4B, respectively. Although electrodeposition has been made by scanning the potential, in a precursor salt solution, from 5 to 10 times between + 0.8 and 0.0 V at 10 mV s^−1^ on ITO (indium tin oxide) electrodes using a Ag/AgCl quasireference/counter electrode [21] and 5 times from + 1.0 to − 0.1 V at 200 mV s^−1^ on HOPG (highly ordered pyrolytic graphite) with Ag/AgCl reference electrode and Pt counter electrode [22], in this case, applying a constant potential for a time was chosen for warranty deposition of Pd from PdNCs released from immunocomplexes. Atoms on the cluster surface could be reduced becoming more catalytic as well as Pd cations that could be on the electrode/electrolyte interface, generating new nucleation and/or growth processes to form fresh active aggregates that increase the analytical signal. In relation to the potential, this has been assessed between − 0.4 and − 0.7 V achieving the highest signal at − 0.6 V, which was chosen for the remainder of the work. Regarding the electrodeposition time, as it increases, the signal increases up to 90 s, time employed as optimum since a good signal is obtained without lengthening the analysis time considerably. On the other hand, since PdNCs catalyse the HER, a higher acid concentration could imply an increase in the reaction rate that will benefit their catalytic action [49]. In this vein, the acid concentration has been evaluated between 0.1 and 1.0 M (Fig. S4C), being 0.75 M HNO_3_, that showed an increase of about 40% in the signal, employed for further studies. Subsequently, a calibration was carried out by LSV using aqueous solutions ranging from 1 to 75 ng g^−1^ of PdNCs in a medium containing 8% SDS to dissolve the MNCs [26].

**Fig. 1:**
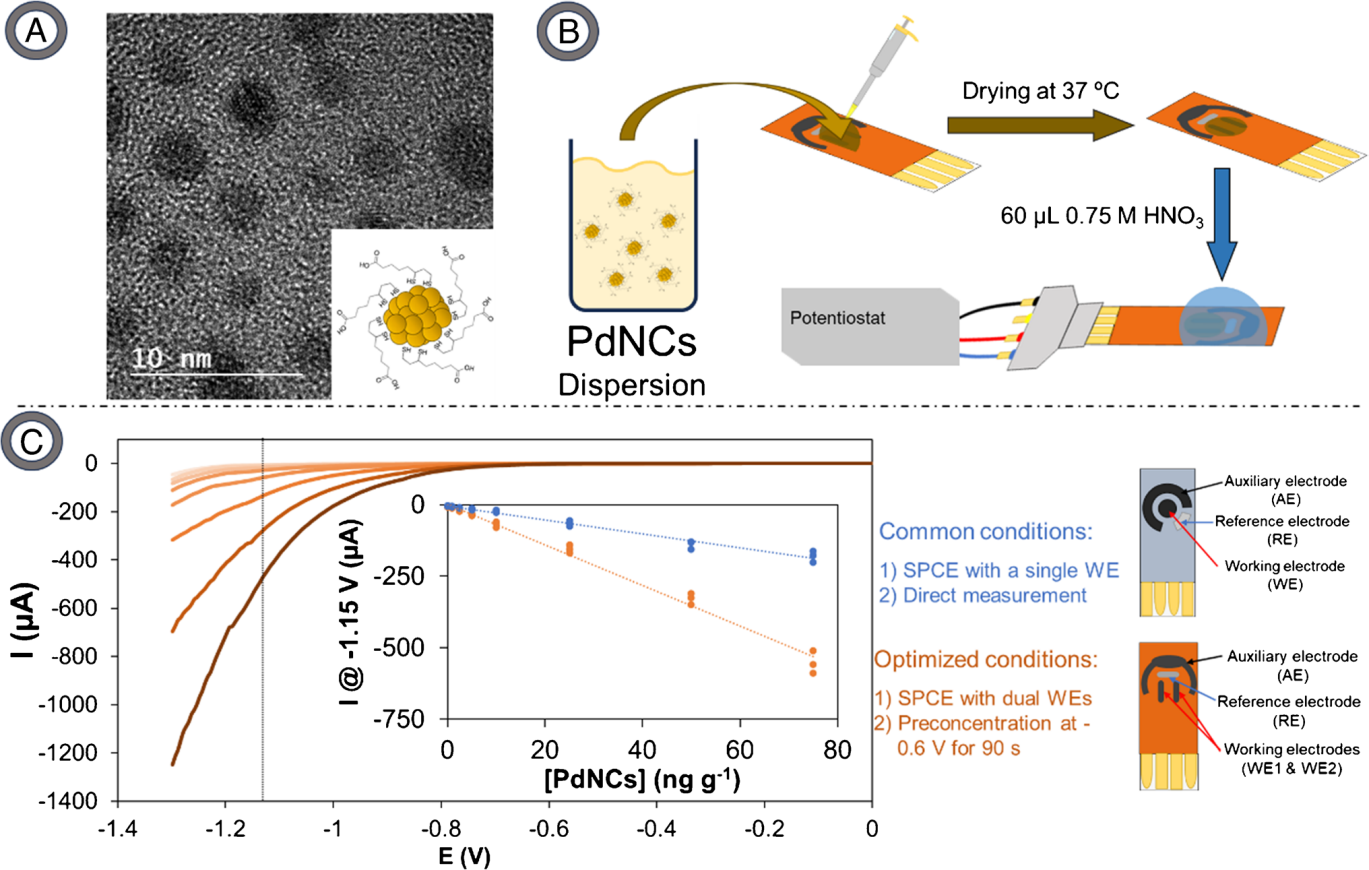
**A** Characterization of PdNCs by HR-TEM together with a drawing of a PdNC. **B** Methodology used to carry out electrochemical measurements. **C** Linear sweep voltammograms obtained after depositing 6 µL of a PdNCs dispersion on the electrode at concentrations between 1 and 75 ng g^−1^ by using dual WE electrodes and electrodeposition at − 0.6 V for 90 s. The inset shows the calibration curve obtained representing the intensity of the current measured at − 1.15 V versus the concentration of PdNCs when using single WE electrode without preconcentration (blue) and dual WE electrodes electrodepositing (black). Three measurements are represented for each concentration

Figure 1A shows an image of the PdNCs obtained by HR-TEM, as well as a representation of a PdNC. Figure 1B also shows a diagram detailing the methodology used to carry out the electrochemical measurements. In addition, Fig. 1C displays the voltammograms (recoded in 0.75 M HNO_3_) obtained for each of the evaluated PdNCs concentrations (without performing any immunoassay) using SPCEs with two WEs and the electrodeposition step (at − 0.6 V for 90 s). Signals recorded with cards containing two WEs were compared with those obtained with traditional one-WE cards. Thus, a drawing of the electrodes used and the experimental conditions employed for each case has been added. Importantly, in the analysis of the results it is possible to represent either (i) the intensity of the current obtained for a fixed potential, or (ii) the potential at which a particular current intensity is reached (Fig. S5). Since taking the current intensity at a fixed potential provided higher analytical signals, this was the analytical signal recorded for all subsequent studies. The inset in Fig. 1C represents visually the current intensity obtained at − 1.15 V plotted against the concentration of PdNCs, in a range of 1–75 ng g^−1^. The results, obtained under optimized conditions, revealed a sensitivity of 7.1 ± 0.2 µA g ng^−1^. The limit of detection (LoD), of 0.5 ng g^−1^ PdNCs, was calculated as the signal corresponding to 3 times the SD of the intercept obtained in the low concentration range (from 1 to 10 considering also the blank). Compared to the values obtained with one-WE card, sensitivity of 2.46 ± 0.09 µA g ng^−1^, and LoD of 0.8 ng g^−1^ PdNCs, it can be said that the optimized methodology allows increasing the sensitivity almost three times, while decreasing the LoD to the half.

Figure S6 shows the two types of SPCEs used (single or dual working electrodes) along with seven replicates carried out with independent SPCEs. For the analysis, the deposition over the WE (or over both WE when dual electrodes were used) of 6 µL of 50 ng g^−1^ of PdNCs and measurement in 0.75 M HNO_3_ (for each type of electrode) were performed. Furthermore, seven replicates using dual SPCEs were also measured while an electrodeposition step was added. SPCEs with two WEs showed greater precision than those with one WE (Relative standard deviation, RSD, of 5.1 vs. 7.2%), although the intensity of the current measured at − 1.15 V was slightly reduced, in absolute value (− 96 ± 5 vs. − 140 ± 10 µA). The enhanced precision may be attributed to the acquisition of two measurements, resulting in the calculation of the average current intensity. On the other hand, the lower intensity is fundamentally due to the smaller area of the working electrode in the double electrode cell. If an electrodeposition step (at − 0.6 V for 90 s) is added, a clear increase in the intensity measured in absolute value (− 330 ± 14 vs. − 96 ± 5 µA) is observed while reproducibility is slightly improved (RSD of 4.2 vs. 5.1%). It should be noted that very small volumes are employed, so part of the error could be due to the imprecision of the micropipette itself (in this case less than 2.5%, i.e., 0.15 in the 6 µL added). Moreover, in Fig. S7, which shows the LSVs for seven measurements on independent SPCEs, the used strategies for improving analytical features improve the analytical signal and the precision of the measurements.

### Competitive immunoassay

**Fig. 2:**
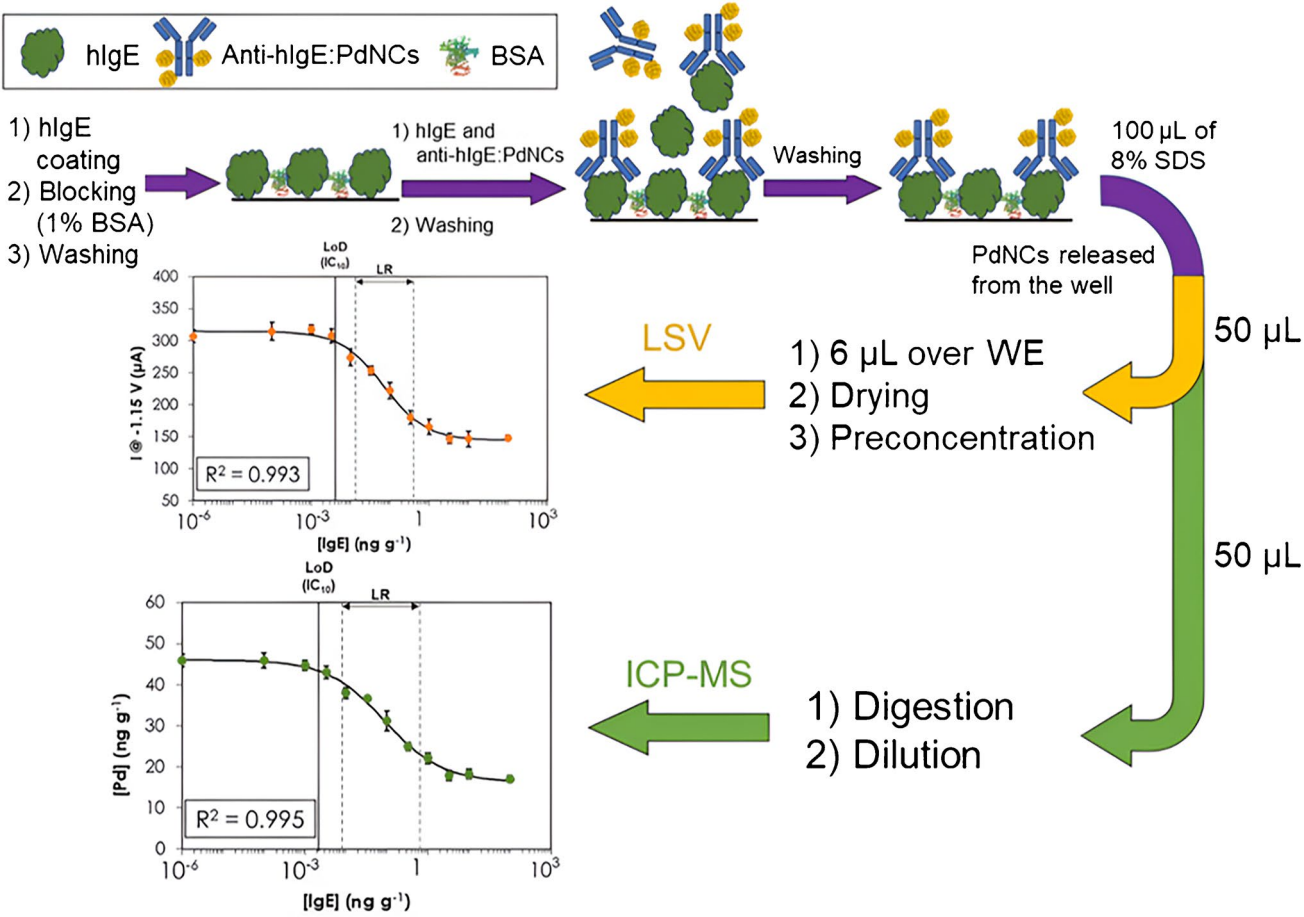
Scheme of the experimental procedure followed for IgE determination. Calibration curves performed using the competitive immunoassay with PdNCs as labels and detection by LSV, measuring the intensity of the current at − 1.15 V (top), and by ICP-MS (bottom). Error bars represent the standard deviation of the mean values of the signal for three independent measurements. LoD stands for limit of detection (determined at IC_10_), while LR indicates the linear range (IC_20_–IC_80_)

There is an initial crucial step, which involves the successful bioconjugation of PdNCs with anti-hIgE Ab, following the procedure outlined in the experimental section. Once this bioconjugation was confirmed, a calibration curve for IgE was performed for concentrations ranging from 10^−5^ to 10^2^ ng g^−1^. A schematic of the immunoassay is included in Fig. 2 for the shake of clarity. Thus, the higher the concentration of IgE in the solution, the lower the number of anti-hIgE:PdNCs immunoprobes bound to the well, and so, the lower the concentration of PdNCs deposited on the electrode surface. Accordingly, the higher the concentration of PdNCs on the surface, the higher the increase in the current measured at − 1.15 V (enhanced catalysis of the HER process). Figure 2 also illustrates the calibration curve obtained using the LSV technique. With the aim of comparing sensitivity and precision of measurements with those obtained with a robust methodology such as elemental MS, a calibration curve was also conducted using ICP-MS (also presented in Fig. 2).

The equations obtained by 4-PL fitting for LSV (Eq. 2) and ICP-MS (Eq. 3) measurements are shown below.

2 $$I\;\left(\upmu A\right)=143.8+ \frac{170.9}{1+{\left(\frac{\left[IgE \right]\;\left(ng\;{g}^{-1}\right)}{7\;{10}^{-5}}\right)}^{0.79}}$$

3 $$[Pd]\;\left(ng\;{g}^{-1}\right)=16.3+ \frac{29.7}{1+{\left(\frac{\left[IgE \right]\;\left(ng\;{g}^{-1}\right)}{9\;{10}^{-5}}\right)}^{0.62}}$$

Remarkably, LSV measurements (using SPCEs with two WEs and the preconcentration step) demonstrated a precision like that achieved with ICP-MS. A 4-PL fit was applied to both curves, obtaining linear ranges on the logarithm scale for x-axis (IC_20_–IC_80_, being IC the inhibitory concentration) from 0.01 to 0.4 ng g^−1^ and from 0.01 to 0.8 ng g^−1^ for LSV and ICP-MS, respectively. The IC_10_ (inhibitory concentration at 10%) is commonly considered the LoD with this type of fitting. Values of 0.004 and 0.003 ng g^−1^ for this parameter were obtained by LSV and ICP-MS, respectively. One of the more reliable methods to calculate the LoD involve the error profile approach [50], which is computed by interpolating the four-parameter fit using the lower asymptote (through a 95% confidence Student *t* test) and inserting the value of parameter *a* (lower limit) into the calibration curve equation using average values. This yielded LoDs of 0.008 and 0.004 ng g^−1^ for LSV and ICP-MS, respectively. Significantly, these detection limits were considerably much lower than those of the commercial kit used for serum sample validation, 5 IU mL^−1^ (*ca*. 12 ng mL^−1^). It should be highlighted that the methodology described for performing LSV has a sensitivity close to this obtained with ICP-MS, which requires expensive equipment and more specialized personnel. Due to this factor, the noteworthy advancements in electrochemical detection of PdNCs enable high-sensitivity analyses to be conducted while upholding low costs and the potential for decentralizing conventional analysis methods.

### Determination of IgE in human fluids

The developed competitive immunoassay based on the use of anti-hIgE:PdNCs immunoprobe was employed to assess IgE levels in various biological fluids, including saliva, tears, nasal exudate, capillary blood, apart from blood serum. The World Health Organization’s ASSURED criteria for miniaturized biomedical devices, which with the advancing technology evolved to the more complete REASSURED criteria, is a comprehensive framework that guides the development and implementation of point-of-care devices. The acronym [51] stands for Real-time connectivity, Ease of specimen collection and Ease of use, Affordable, Sensitive, Specific, User-friendly, Rapid, and robust, Equipment-free, and Deliverable to end-users. This criterion ensures that the devices are accessible, efficient, and effective in various settings, particularly in low-resource environments. Considering this criterion, the immunoelectrochemical approach here developed was applied to samples that require less invasive (saliva, tears, nasal exudate, and capillary blood) extraction methods that these used for commonblood serum sample.

From the human samples described, it has been found that the optimal way to collect salivary fluid was in an unstimulated manner, obtaining higher IgE protein concentration [52], although this does not seem to be the case for other proteins [53]. On the other hand, tears are usually collected by means of Schirmer test strips, ophthalmic sponges, or microcapillary tubes, being the latter the most accepted because it produces the least reflex tearing, thus avoiding the risk of involuntary dilution of the sample [54]. Finally, the best way to collect nasal exudate is by absorption on swabs, which has been shown to be the most reproducible in the scientific literature [54]. In the case of capillary blood, collection is usually done by pricking a finger, collecting the blood on paper or in a tube (like in this case) [55]. There are also devices that allow extraction to be carried out in a single stage, in such a way that the extraction is carried out more quickly and reproducibly [56]. The specific treatment performed in this work for each type of sample is summarized in the ESM. To calculate the concentration of the samples, the signal obtained for that sample (intensity of the current measured at − 1.15 V) is interpolated within the 4-PL curve, which returns an IgE concentration in the immunoassay. To obtain the concentration of the sample, it is necessary to undo the dilutions.

**Fig. 3:**
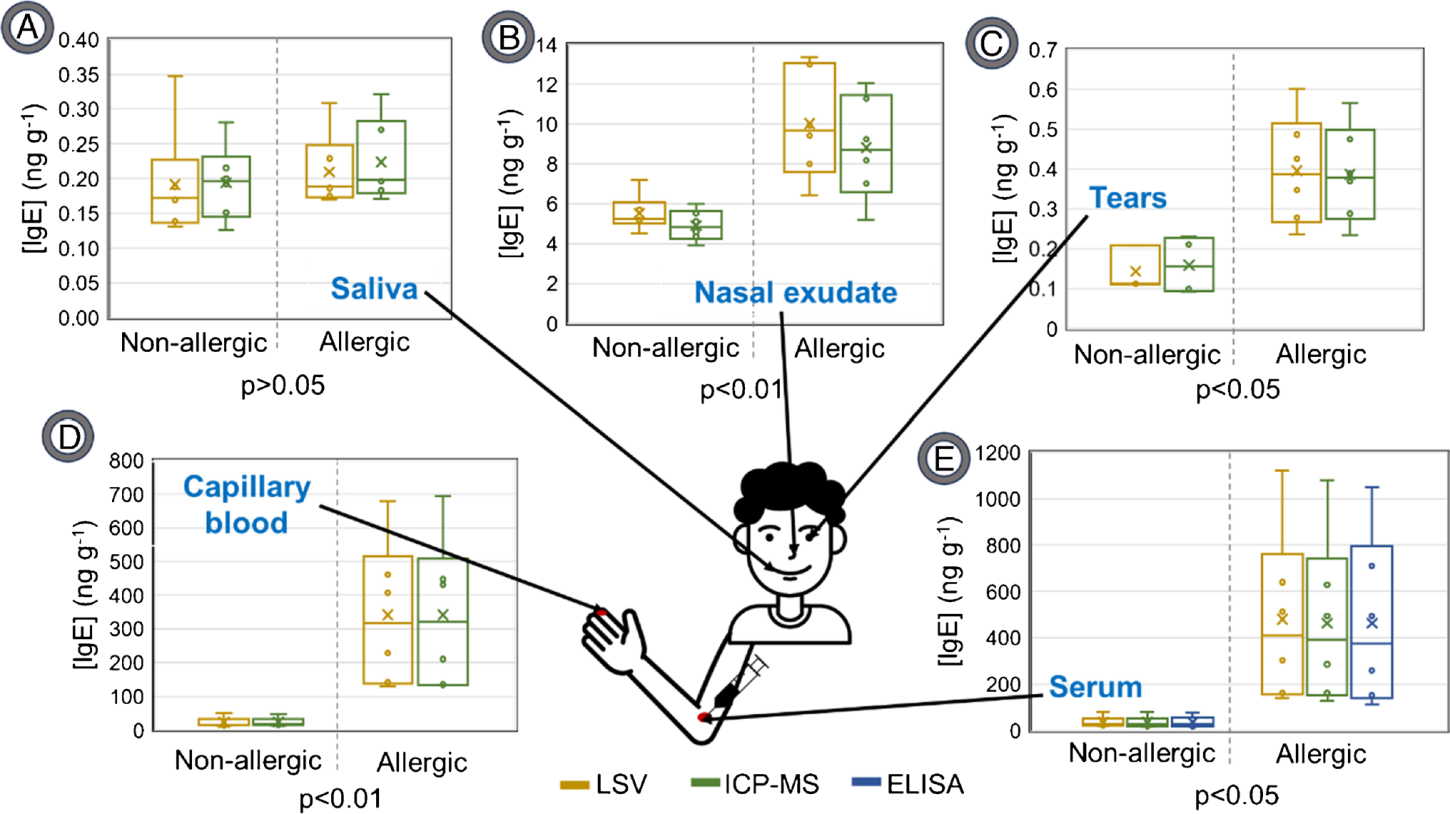
Box and whisker plots for IgE concentration determined in the different human fluids analysed: saliva (**A**), nasal exudate (**B**), tears (**C**), capillary blood (**D**), and serum (**E**). LSV, ICP-MS, and commercial ELISA measurements are shown in yellow, green, and blue, respectively. The average values of the six samples analysed for each biological fluid are shown in each case, except for the tears of non-allergic people, where only the 3 above the LoD are shown

Figure 3 showcases the IgE concentrations found in the analysed samples determined by LSV, compared to the results obtained for the same samples analysed by ICP-MS, and also by a commercial kit ELISA with spectrophotometry detection in the case of serum samples. Notably, it seems that there are no significant variations in the values obtained when different detection techniques (Table S2) are employed. When examining serum samples, there are no significant differences observed between the developed competitive immunoassay (with both LSV and ICP-MS detection) and the commercial ELISA kit for allergic individuals, with *p* values greater than 0.93 for LSV and 0.99 for ICP-MS. Similarly, for non-allergic individuals, *p* values exceed 0.99 for LSV and 0.94 for ICP-MS.

However, our maximum interest is in samples that could be taken in a minimally invasive way. An in-depth analysis of Fig. 3 reveals noticeable differences in IgE concentration between allergic and non-allergic individuals, particularly for serum and capillary blood samples (*p* values 0.015 and 0.0046 for LSV, respectively). Moreover, significant differences (*p* value 0.0052 and 0.019) are observed for nasal exudate and tear samples. However, it is worth noting that due to the low sample volume extracted for tears, combined with their low IgE concentration, values for three samples from non-allergic individuals were undetectable by both techniques. Conversely, the IgE concentration in saliva does not appear to be significantly affected by the presence of allergic rhinitis (*p* value 0.635).

Based on these findings, we can infer that there are significant differences in IgE concentration of individuals with allergic rhinitis, particularly for the analysis of nasal exudate, tears, capillary blood, and serum blood samples. These samples can be collected in a non- or minimally-invasive way and can be obtained quickly, with capillary blood extraction being particularly appealing due to its speed and suitability for patients at all ages and physical conditions. Table I reveals some correlation between the IgE concentration found in samples, for each individual, when LSV detection was performed.

**Table 1:** IgE concentration detected by LSV (presented as mean ± SD, *n* = 3) determined for each of the examined samples

	Sample (*n*)	Nasal exudate (ng g^−1^)	Saliva (ng g^−1^)	Tears (ng g^−1^)	Capillary blood (ng g^−1^)	Serum (ng g^−1^)
Non-allergic	1	5.2 ± 0.8	0.35 ± 0.09	< LoD	15 ± 2	20 ± 3
	2	5.7 ± 0.8	0.17 ± 0.03	< LoD	15 ± 2	30 ± 2
	3	5.3 ± 0.4	0.19 ± 0.04	0.11 ± 0.04	14 ± 3	27 ± 2
	4	4.5 ± 0.7	0.17 ± 0.02	0.21 ± 0.04	13 ± 1	21 ± 2
	5	7 ± 1	0.13 ± 0.02	< LoD	49 ± 6	80 ± 8
	6	5 ± 1	0.14 ± 0.02	0.11 ± 0.03	26 ± 4	44 ± 4
Allergic rhinitis	1	10 ± 1	0.18 ± 0.02	0.60 ± 0.04	130 ± 10	160 ± 20
	2	8 ± 1	0.17 ± 0.03	0.35 ± 0.03	270 ± 20	300 ± 30
	3	13 ± 1	0.31 ± 0.02	0.43 ± 0.06	700 ± 100	1100 ± 200
	4	9 ± 2	0.19 ± 0.01	0.24 ± 0.03	470 ± 90	640 ± 90
	5	7 ± 1	0.23 ± 0.03	0.28 ± 0.04	140 ± 20	140 ± 10
	6	13 ± 1	0.19 ± 0.02	0.49 ± 0.05	410 ± 50	510 ± 60

**Fig. 4:**
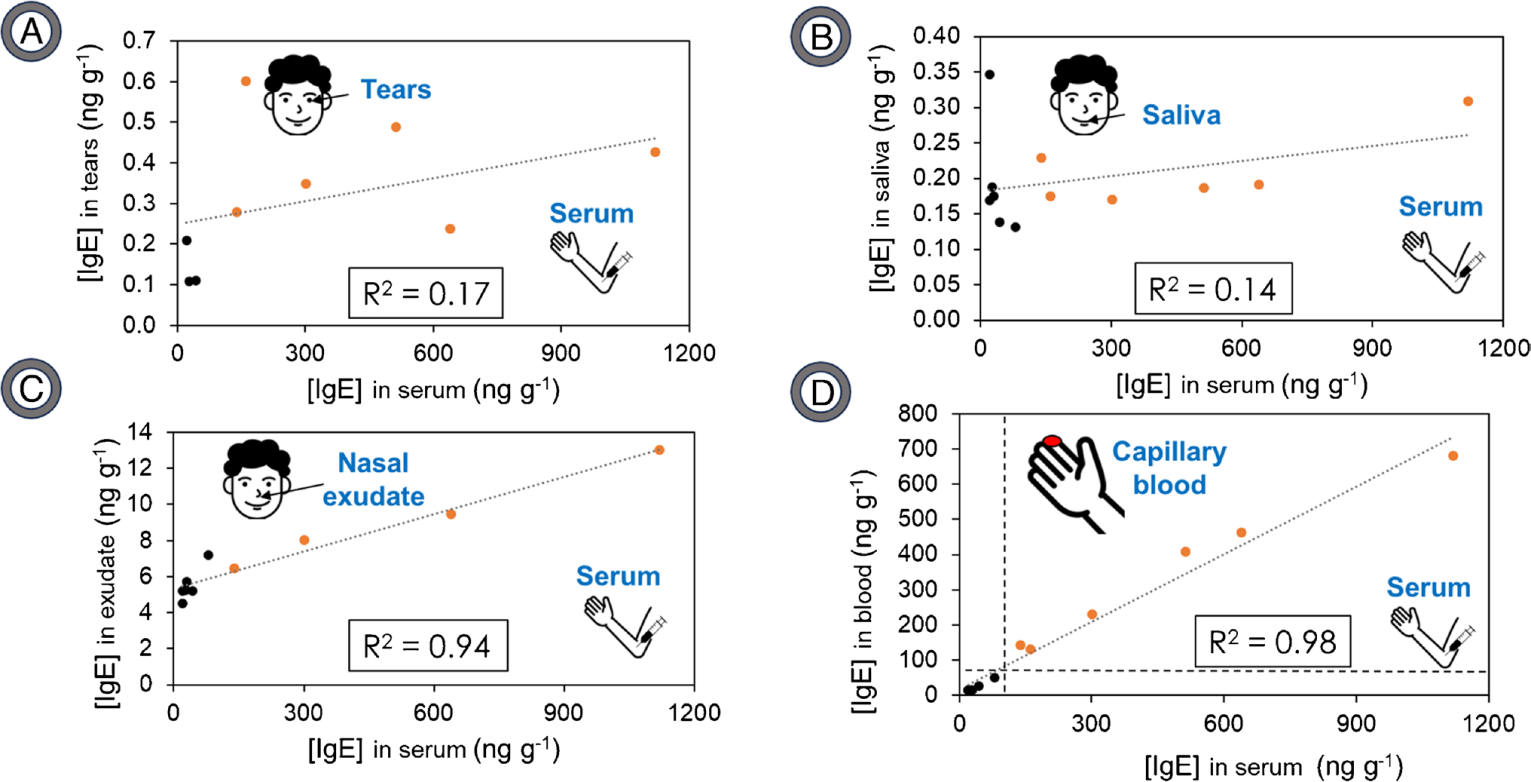
Graphical representation of IgE concentration in tears (**A**), saliva (**B**), nasal exudate (**C**), and capillary blood (**D**) versus IgE concentration found in serum for the same donor determined by LSV. Orange for allergic and black for non-allergic individuals. The vertical and horizontal dashed lines show possible cutoff levels for serum (100 ng g^−1^) and capillary blood (60 ng g^−1^) samples

Analogous results were found for detection by ICP-MS (Table S3). To illustrate these differences more effectively, Fig. 4 displays the concentration obtained for each analysed sample (saliva, nasal exudate, tears, and capillary blood) versus the IgE concentration in serum blood, as detected by LSV.

Notably, there does not appear to be a substantial linear correlation between the IgE concentration in saliva or tears and the corresponding levels in serum blood (*R*^2^ of 0.14 and 0.17, respectively). An analysis of the data reveals a certain correlation between the values obtained from nasal exudate samples and those from serum blood (*R*^2^ of 0.81). This becomes even more marked when data from two individuals who have been identified as allergic are excluded (*R*^2^ of 0.95). Importantly, the IgE concentration in capillary blood is highly correlated with the concentration in serum blood (*R*^2^ of 0.99). This relationship could be of great interest because it is much simpler to extract capillary blood. Likewise, it does not seem that tears are very appropriate due to the difficulty involved in obtaining them in a reproducible manner, requiring some expertise in the sampling so that the patient does not cry during the extraction. It is also important to note that not everyone can adequately produce tears, as people with dry eyes would not be suitable for this type of sampling. On the contrary, it seems that the nasal exudate is a promising sample since it is simple to extract and there is an acceptable correlation with serum. In this way, and summarising, capillary blood and nasal exudate seem to be the most appropriate samples. Both fluids can be extracted quickly, easily, and by anyone without the need for specific equipment or qualified personnel.

Finally, when comparing the results obtained in this study with those obtained for another protein (glial fibrillary acidic protein, a stroke biomarker [33]) also following a competitive immunoassay with PdNCs, it is evident that there has been a significant improvement in electrochemical measurements by LSV, particularly in terms of precision. These enhancements, coupled with the use of non-invasive samples, pave the way for the development of electroanalytical methodologies for decentralized application. The use of capillary blood is especially interesting because of its strong correlation with serum, opening the possibility of replacing it for IgE monitoring. This would simplify sample collection, making it feasible for untrained personnel.

## Conclusions

Low-cost portable devices and non-invasive sampling allow decentralization of analysis. However, methodologies must provide reliable measurements with adequate sensitivity and precision. This has been demonstrated for IgE determination in minimally invasive samples (tears, nasal exudate, saliva, capillary blood, and serum blood) from individuals without allergic pathologies and those with allergic rhinitis. The use of screen-printed electrochemical cells with two working electrodes led to a notable improvement in the precision of the measurements, based on the catalytical activity of PdNCs. Sensitivity and LoD were highly improved by freshly electrodepositing PdNCs just before the measurement. The developed electrochemical approach was tested by performing competitive immunoassays using PdNCs as a catalytic label to determine IgE. When this approach is compared to the centralized and robust ICP-MS technique, it becomes evident that the developed electrochemical measurements exhibit adequate accuracy and reproducibility for IgE determination. This is supported by the similarity in results obtained using both detection strategies for the same samples. Concerning the different human fluids evaluated for the allergic and non-allergic donors, significant differences were found between both groups for all types of samples analysed (except for saliva). Linear correlation between the IgE concentration found in serum and capillary blood and, also, nasal exudate was observed.

Combining the analytical performances achieved for LSV analysis with the ability to conduct IgE determination in non-invasive and easy-to-extract samples, such as capillary blood, the proposed methodology enables adaptation to decentralized analysis that could be performed by untrained personnel in extrahospitalary settings. The electrochemical methodology is much simpler and sensitive and requires lower cost equipment than this currently used (commercial ELISA). Nonetheless, the long duration of the competitive immunoassay is a limitation that should be addressed in further works, possibly by adopting faster methods, such as vertical flow immunoassays, that fit perfectly with electrochemical detection. Another interesting possibility consists in using PdNCs that are modified to present higher catalytic activity on HER. We consider particularly attractive the case of bimetallic NCs [57], which have presented a significantly higher catalytic activity than monometallic NCs for HER [58].

## Supplementary information

Below is the link to the electronic supplementary material.

Supplementary file1 (DOCX 290 KB)

## Data Availability

The dataset supporting this study will be available at the Repository of the University of Oviedo and this of the Principality of Asturias.
