# Supplementary material for: Immunoelectrochemical assessment of human IgE in non-invasive samples of allergic individuals using PdNCs-labelled antibodies
Source: Mikrochim Acta. 2025 Mar 18;192(4):237. doi: 10.1007/s00604-025-07083-3 (PMC11920355; doi:10.1007/s00604-025-07083-3)
Supplement: Supplementary file 1 — Supplementary file1 (DOCX 290 KB) [file 604_2025_7083_MOESM1_ESM.docx]

Electronic Supporting Material on the Analytical Chemistry entitled

**Immunoelectrochemical assessment of human IgE in non-invasive samples of allergic individuals using PdNCs-labelled antibodies**

Alejandro Rodríguez-Penedo, Estefanía Costa-Rama, Rosario Pereiro, Beatriz Fernández,^*^ M. Teresa Fernández-Abedul^*^

Department of Physical and Analytical Chemistry, University of Oviedo, Julian Clavería 8, 33006 Oviedo, Spain

*Corresponding authors email addresses:

[mtfernandeza@uniovi.es](mailto:mtfernandeza@uniovi.es) & [fernandezbeatriz@uniovi.es](mailto:fernandezbeatriz@uniovi.es)

**DESCRIPTION OF THE ELECTRONIC SUPPLEMENTARY MATERIAL**

Details of the Experimental Section and Results and Discussion section are shown in this Electronic Supplementary Material (ESM), which consists of the following points:

- A section indicating the procedure for collection and treatment for each of the sample types tested as well as the informed consent.
- Seven figures related to the catalytic activity of the PdNCs, their bioconjugation and the competitive immunoassay.
- Three tables showing the experimental parameters for ICP-MS analysis and the concentration obtained for each of the samples analyzed by ICP-MS and by means of a commercial ELISA.

***Experimental Section: Collection and Processing of Biological Samples***

- **Saliva.** Before initiating saliva collection, volunteers were instructed to rinse their mouths for 3 min with tap water followed by 30 s with sterilized water. Subsequently, unstimulated saliva was collected directly into sterile tubes, which were immediately placed on ice and centrifuged at 1800 g for 10 min. Finally, the supernatant was collected, aliquoted, and stored at -80°C for future use. Before performing the immunoassay, and considering the low concentration in saliva, a 1:4 dilution with Tris-HCl buffer was performed.
- **Tears.** A volume of 10 μL of tear fluid was extracted using calibrated glass microcapillary tubes (Blaubrand intraMark). To perform this, the patient's eyelid is gently opened, and the capillary is placed horizontally in relation to the blinking motion. It is important to note that the fluid around the eye should be collected, and the sample is not valid if the patient starts tearing up. The collected tears were stored in labelled 500 μL tubes and stored at -80°C. Before performing the immunoassay, and considering the low sample volume extracted, a 1:16 dilution was made with Tris-HCl buffer to obtain 160 µL, required to perform three replicates.
- **Nasal exudate.** Nasopharyngeal swab (Nest Biotechnology) and tube (containing 320 μL of 10 mM Tris-HCl pH 7.0) employed for collection are exactly weighed before sampling. Then, nasal exudate is extracted using a swab inserted into the nasal passage, making gentle circular motions for about 5 s in each nostril. Subsequently, the swab was added to the prepared Tris-HCl solution, and the weight was recorded. To extract the nasal exudate, the mixture was vigorously shaken for 1 min, and upon removing the swab from the solution, it was pressed repeatedly against the walls and the lid of the tube. Finally, the samples were frozen at -80°C for subsequent use. For performing the immunoassay, extra dilutions were not necessary. The estimated final dilution is ca. 1:32, depending on the volume sample
- **Capillary blood**. A puncture with a lancet is made while applying pressure to the wound to obtain a blood droplet. Subsequently, 5 μL were collected using a micropipette. The collected blood was added to a tube containing 320 μL of 10 mM Tris-HCl pH 7.0 maintained at 4°C, and it was immediately centrifuged at 1800 g for 10 min. Finally, the supernatant was separated and stored at -80°C for future use. For performing the immunoassay, a 1:3 dilution with Tris-HCl buffer was made for samples coming from non-allergic patients and 1:20 for allergic patients, due to the significant differences between the two groups. Considering the previous dilution made during sample treatment, the final dilution for capillary blood samples would be 1:192 for non-allergic patients and 1:1280 for allergic patients.
- **Blood serum.** In all cases, blood was collected in 5 mL Z Serum Sep Clot Activator tubes coated with microscopic silica particles, which activate the coagulation process (Vacuette, Madrid, Spain). The tubes were centrifuged at 1800 g for 18 min at 4 ºC, and the supernatant (serum) was stored at −80 ºC until use. For performing the immunoassay, a 1:200 dilution with Tris-HCl buffer was performed for samples from non-allergic patients and 1:2000 for allergy patients, mainly due to the wide variability in the concentration of hIgE both between the two groups and within the group of allergic individuals.

***Experimental Section: Informed Consent for Sample Collection***

**Project: Determination of IgE in samples of biological origin from:**

**[ ] Controls [ ] Allergy [ ] Other _________**

**Date: ___________**

**Mr./Ms.___________________________, _____ years old, residing in _____________________ and ID n._____________**

**Sample object of study:**

**[ ] Blood [ ] Capillary blood [ ] Tear [ ] Nasal exudate [ ] Saliva [ ] Other _________**

**I DECLARE:**

It has been EXPLAINED to me by the researcher Mr./Ms. ____________________________

member number _____________________

and I have UNDERSTOOD that:

1-. The samples mentioned above are going to be extracted from me to include them in a study that seeks to develop a methodology based on analytical immunoassays that allows the determination of IgE in different types of biological samples (saliva, tears, nasal exudate, blood serum extracted from blood intravenous and blood serum extracted from capillary blood). The immunoassays developed will be based on the use of antibodies labeled with palladium nanoclusters (PdNCs) and will be measured by elemental mass spectrometry and electrochemical techniques.

2-. The purpose of this study is to develop a methodology that allows IgE to be detected through noninvasive tests.

3-. The research consists of the analysis of my sample for the quantification of IgE related to allergy.

4-. It is possible that no conclusive results about the disease may be derived from this study due to the complexity of this pathology. I understand that this study is a collaboration with the progression of science and no benefit is derived from it for my pathology nor does it harm me if the sample is used for this purpose.

5-. These studies can last for months, until the result is obtained.

6-. The research service will maintain confidentiality about the results of the study.

7-. The sample will not be used for any other research, and in that case a new informed consent will be requested from the patient.

At any time, I can refuse to continue with this study, without said resignation entailing any prejudice in the follow-up or treatment that the researchers at the Fernández-Vega Ophthalmological Institute and the Ophthalmological Research Foundation will carry out for my disease.

**I ACCEPT and GIVE MY CONSENT** for the above sample to be used to participate in said study.

**Patient's signature**

**Physician's signature explaining informed consent**

**Witness Signature**

***FIGURES***

***Experimental Section: Synthesis of PdNCs and Labelling of Anti-hIgE***

***
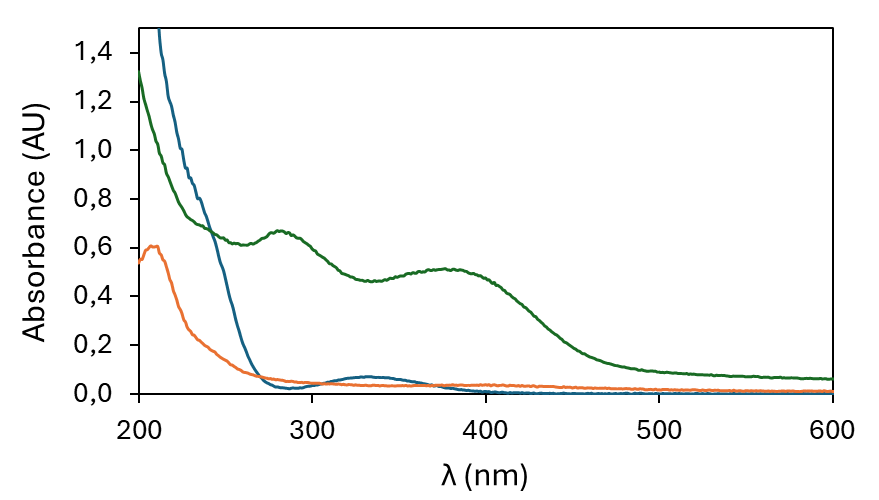
***

**Figure S1**. Absorbance spectrum obtained for synthesis: blank without K_2_PdCl_4_ (blue), K_2_PdCl_4_ (orange) and PdNCs (green). In all cases, a 1:8 dilution with ultrapure water was performed.


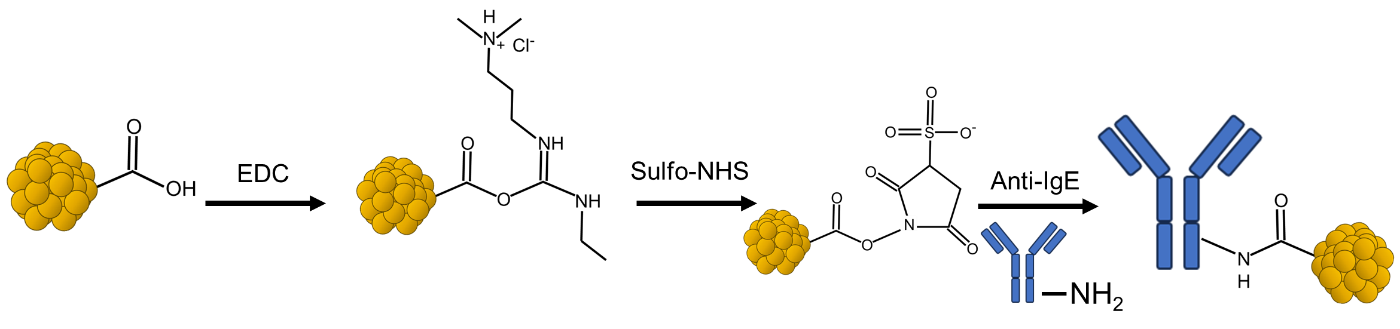


**Figure S2**. Schematic representation of the carbodiimide method used to bioconjugate the PdNCs to the antibodies.


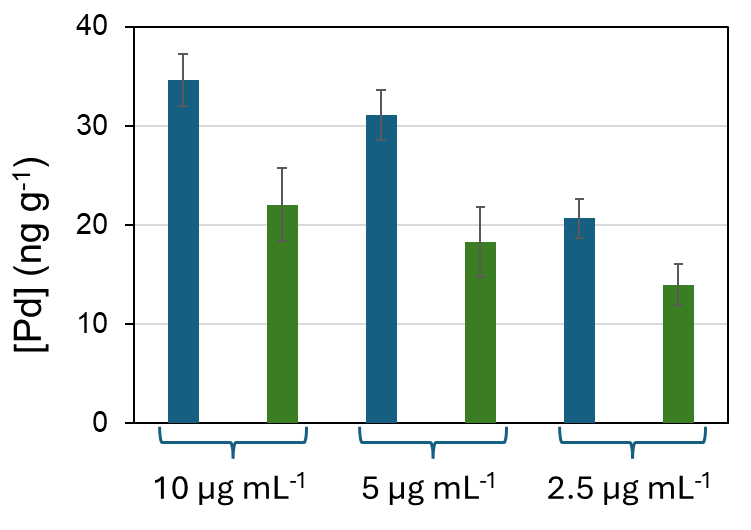


**Figure S3**. Concentration of PdNCs recovered from the well for immunoassays performed by adding 100 µL of a 10, 5 and 2.5 µg mL^-1^ concentration of hIgE. The blue bars show results when 10^-3^ ng g^-1^ of hIgE was added in the competitive format along with the immunoprobe. The results shown in green are for the addition of 10 ng g^-1^ of hIgE.

The figure shows a reduction of the signal with decreasing concentration of hIgE used in the well both when using 10^-3^ ng g^-1^ or 10 ng g^-1^ of hIgE in the competitive format, being especially pronounced for the concentration of 2.5 µg mL^-1^. On the other hand, the difference between the concentration of Pd recovered from the well when using 5 or 10 µg mL^-1^ is not significant. Likewise, the background signal (when using 10 ng g^-1^ of hIgE) is smaller when using 5 µg mL^-1^, which should help to slightly improve precision. This concentration was then chosen for the remainder of the work.

***Results and Discussion: Optimization of the Electrochemical Measurements***

1.
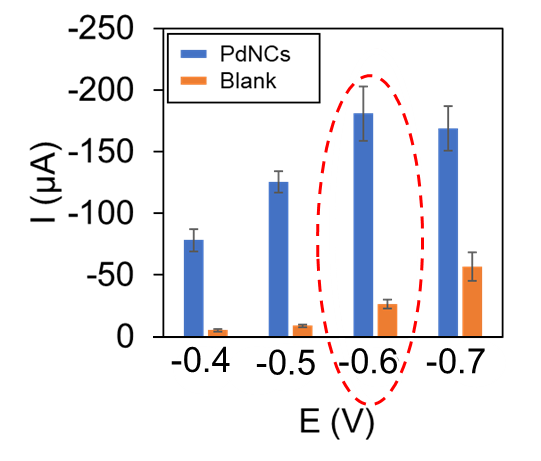

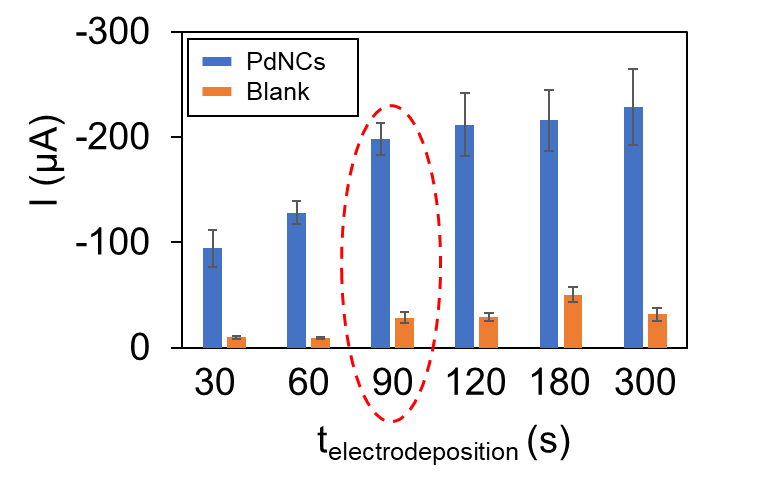
Electrodeposition potential B) Electrodeposition time
2.
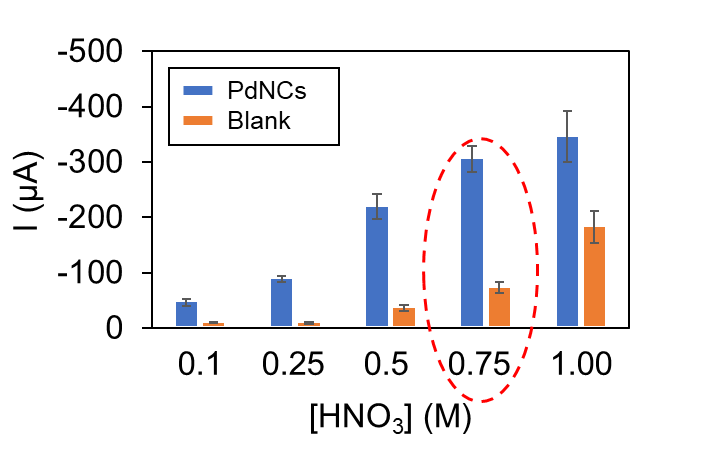
Concentration of nitric acid

**Figure S4**. Optimization of the electrodeposition potential (A) and time (B), and the HNO_3_ concentration (C), using in all cases 50 ng g^-1^ of PdNCs. LSV was employed, measuring the current intensity at -1.15 V. Uncertainties represent the standard deviation of the mean of three measurements.

***Results and Discussion: Measurement of the potential or current difference***

**
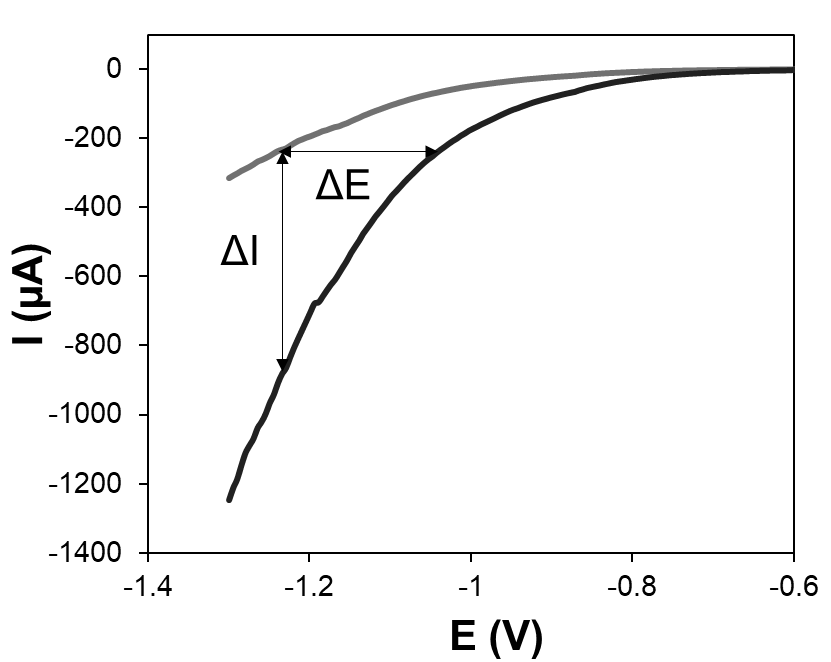
**

**Figure S5.** Example of how the difference between two voltammograms can be quantified by measuring the current at a given potential or the potential at a fixed current.

***Results and Discussion: Signals obtained for single/dual WEs with/without electrodeposition***


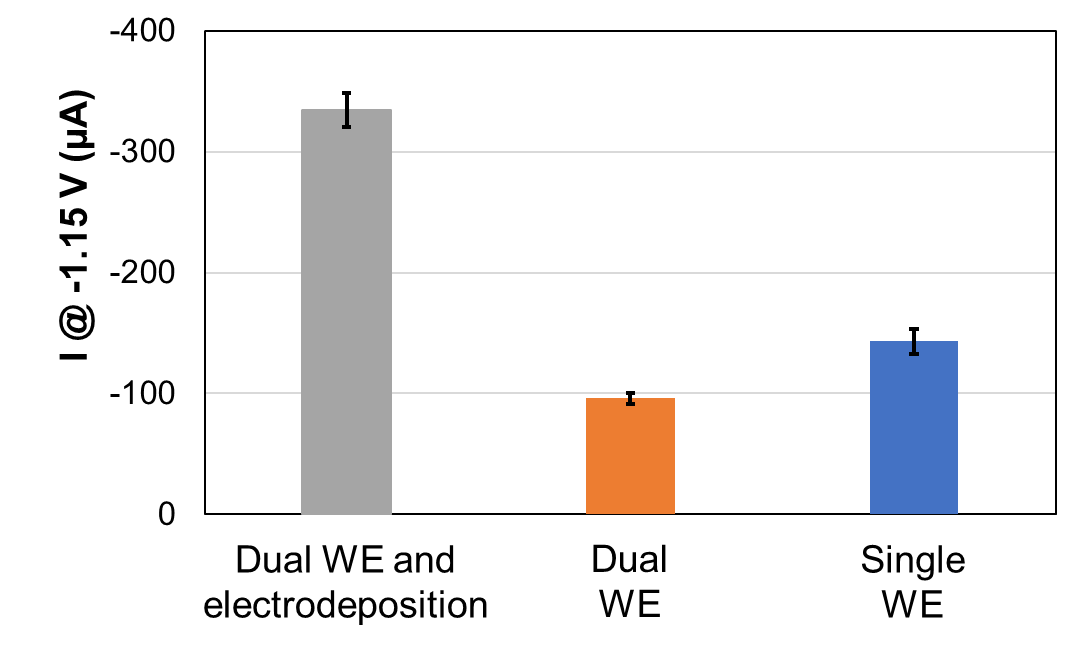


**Figure S6.** Intensity of the current obtained at -1.15 V for 7 replicates performed with independent electrochemical cells, either with single WE (blue), dual WE (orange), or with dual WE after adding an electrodeposition stage (grey). In all cases, solutions with 50 ng g^-1^ of PdNCs in 0.75 M of HNO_3_ were used.


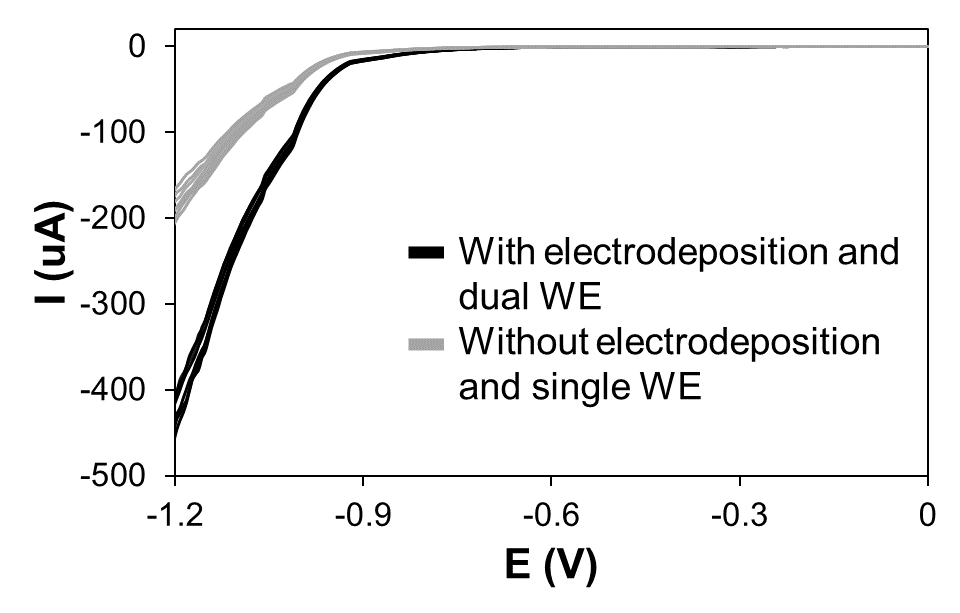


**Figure S7.** Voltammograms performed with SPCE with a single WE and no electrodeposition (grey) and with SPCE with two WEs, electrodepositing at -0.6 V during 90 s (black). In all cases, solutions with 50 ng g^-1^ of PdNCs in 0.75 M of HNO_3_ are used.

***TABLES***

***Experimental Section. Instrumentation.***

**Table S1**. Optimized experimental parameters under which the ICP-MS measurements were conducted.

| Parameter | Values |
| --- | --- |
| ICP RF power (W) | 1550 |
| Plasma gas flow (L min^-1^) | 15 |
| Make up gas flow (L min^-1^) | 0.10 |
| Nebulizer gas flow (L min^-1^) | 1.07 |
| Isotopes (m/z) | ^103^Rh,^105^Pd,^106^Pd |
| Dwell time (ms) | 500 |

***Results and discussion: Determination of IgE in biological samples***

**Table S2.** Comparison of results obtained for IgE concentration in serum blood by electrochemical detection (LSV), ICP-MS, and the commercial immunoassay ELISA. Results are expressed as mean ± SD for three replicates.

| **Sample No.** | **[IgE] by LSV**  **(ng g^-1^)** | **[IgE] by ICP-MS**  **(ng g^-1^)** | **[IgE] by ELISA**  **(ng g^-1^)** |  |
| --- | --- | --- | --- | --- |
| **Non-allergic** | 1 | 20 ± 3 | 20 ± 3 | 18 ± 1 |
|  | 2 | 30 ± 2 | 28 ± 3 | 30 ± 2 |
|  | 3 | 27 ± 2 | 26 ± 1 | 30 ± 2 |
|  | 4 | 21 ± 2 | 19 ± 1 | 18 ± 2 |
|  | 5 | 80 ± 8 | 80 ± 10 | 79 ± 8 |
|  | 6 | 44 ± 4 | 43 ± 3 | 47 ± 2 |
| **Allergic rhinitis** | 1 | 160 ± 20 | 160 ± 10 | 157 ± 5 |
|  | 2 | 300 ± 30 | 290 ± 20 | 264 ± 9 |
|  | 3 | 1100 ± 200 | 1110 ± 200 | 1050 ± 50 |
|  | 4 | 640 ± 90 | 630 ± 90 | 690 ± 30 |
|  | 5 | 140 ± 10 | 130 ± 10 | 109 ± 4 |
|  | 6 | 510 ± 60 | 490 ± 60 | 510 ± 20 |

**Table S3.** IgE concentration (expressed as mean ± SD; n=3) obtained by ICP-MS for each of the five human fluid samples tested (tears, saliva, nasal exudate, serum, and capillary blood).

| **Sample No.** | **Nasal exudate**  **(ng g^-1^)** | **Saliva**  **(ng g^-1^)** | **Tears**  **(ng g^-1^)** | **Capillary blood**  **(ng g^-1^)** | **Serum**  **(ng g^-1^)** |  |
| --- | --- | --- | --- | --- | --- | --- |
| **Non-allergic** | 1 | 5.5 ± 0.4 | 0.28 ±0.04 | <LoD | 16 ± 1 | 20 ± 3 |
|  | 2 | 5.1 ± 0.7 | 0.20 ±0.03 | <LoD | 16 ± 2 | 28 ± 3 |
|  | 3 | 4.3 ± 0.5 | 0.22 ±0.02 | 0.09 ± 0.02 | 18 ± 4 | 26 ± 1 |
|  | 4 | 3.9 ± 0.5 | 0.19 ±0.02 | 0.21 ± 0.05 | 16 ± 2 | 19 ± 1 |
|  | 5 | 6.0 ± 0.8 | 0.15 ±0.02 | <LoD | 48 ± 3 | 80 ± 10 |
|  | 6 | 4.6 ± 0.9 | 0.13 ±0.03 | 0.23 ± 0.03 | 28 ± 6 | 43 ± 3 |
| **Allergic rhinitis** | 1 | 8.2 ± 0.8 | 0.20 ± 0.02 | 0.57 ± 0.01 | 133 ± 6 | 160 ± 10 |
|  | 2 | 7.0 ± 0.8 | 0.18 ± 0.03 | 0.29 ± 0.02 | 210 ± 10 | 290 ± 20 |
|  | 3 | 12 ± 1 | 0.32 ± 0.05 | 0.39 ± 0.06 | 690 ± 70 | 1110 ± 200 |
|  | 4 | 9 ± 2 | 0.20 ± 0.02 | 0.23 ± 0.02 | 450 ± 60 | 630 ± 90 |
|  | 5 | 5.2 ± 0.3 | 0.27 ± 0.03 | 0.37 ± 0.05 | 140 ± 10 | 130 ± 10 |
|  | 6 | 11.3 ± 0.8 | 0.17 ± 0.01 | 0.48 ± 0.02 | 430 ± 50 | 490 ± 60 |
